# Guidance on guideline adherence

**DOI:** 10.1007/s12471-015-0670-0

**Published:** 2015-03-10

**Authors:** R.J.G. Peters

**Affiliations:** Department of Cardiology, F3-326, Academic Medical Center, PO Box 22660, 1100 DD Amsterdam, The Netherlands

Recurrent ischaemic events are common in patients discharged after an acute coronary syndrome (ACS). In a large observational study in 16,321 patients, 20 % were rehospitalised and 18 % of the men and 23 % of the women older than 40 years died in the first year following discharge [1].

Adequate secondary prevention after ACS may save at least as many lives as treatment provided during the acute phase [2, 3]. Equally important as preventive medication, improvements in lifestyle-related risk factors will result in significant risk reductions. In the OASIS-5 trial, patients with non-ST-segment elevation (STE) ACS were encouraged to adhere to a healthy diet, regular physical activity and smoking cessation. Patients who adhered to both diet and exercise had a relative risk reduction (RRR) of 54 % for myocardial infarction (MI), stroke or death (odds ratio (OR) 0.46; 95 % confidence interval (CI) 0.38–0.57; *p* = 0.0001), and for those who gave up smoking a RRR of 43 % for MI (OR 0.57; 95 % CI 0.36–0.89;p<0.0145) [4].

Written guidelines in medicine have been in use for thousands of years. However, their nature and status have changed substantially in recent years. Most importantly, they are now based primarily on evidence, as opposed to authority or tradition, and they now have a major legal and financial impact. In spite of substantial experience with guidelines in medicine, important issues remain, ranging from how to select members of the writing group to challenges with implementation in clinical practice.

The paper in this issue by Tra et al. [5] addresses implementation. The design of this study has been published separately [6]. The authors report a large retrospective observation on adherence to European Society of Cardiology (ESC) guidelines on preventive medication after ACSs [7]. In the 13 (out of 91) medical centres in the Netherlands that were randomly selected for the analysis, overall adherence to guideline-recommended preventive medication was acceptable, with complete adherence to the five recommended classes of drugs in almost 70 % of the 2471 patients in the analysis. This proportion may in fact be underestimated, for several reasons. First, at discharge not all long-term medication may have been initiated. In particular, blood pressure lowering medication should be titrated to blood pressure and to patient tolerance. Gradual increases in drug therapy would be prudent, particularly in the 50 % of the patients in the study who were not classified as hypertensive. Second, no information on left ventricular function is presented. Patients with preserved ejection fraction and no other indication for renin-angiotensin-aldosterone system (RAAS) blocking agents (such as diabetes) may be managed without RAAS blockers, as per ESC guidelines. Third, angiotensin receptor-blocking agents may have been used in patients who do not tolerate ACE inhibitors. This was not recorded in the study, as the authors report, but should be considered as adherence to the guidelines.

Several limitations need to be considered. The proportion of patients who reached the target levels for blood pressure, low-density lipoprotein cholesterol, glucose or HbA1c was not reported. Mere initiation of medication is not sufficient for achieving the full potential of prevention. Second, lifestyle risk factors are not addressed in the analysis. Clearly, smoking, physical activity and weight management should be addressed, even during the hospital phase. With no information in these aspects, even complete adherence to medication should not be seen as evidence of good overall adherence to the guideline. Third, oral anticoagulants are not considered in the analysis, although they would clearly impact on preventive drug selection. Fourth, the prescription of drugs that are in fact not indicated or contraindicated (e.g. beta blockers in patients with severe asthma) should have been part of the analysis since this may be classified as non-adherence.

Although the findings in the study may therefore not be quantitatively accurate, they do provide a useful estimate. They may be used to rank medical centres according to adherence and to selectively address those where improvements would be most desirable. This may be accomplished by feedback and benchmarking. Advice could be tailored, depending on specific issues per centre.

The involvement of nurses and nurse-led clinics may be considered. Important improvements in guideline adherence have been documented in randomised trials [8]. Preventive medicine requires discipline from healthcare providers, and this may not be a field where physicians outperform others.
